# Porcine epidemic diarrhea virus (PEDV) co-infection induced chlamydial persistence/stress does not require viral replication

**DOI:** 10.3389/fcimb.2014.00020

**Published:** 2014-03-13

**Authors:** Robert V. Schoborg, Nicole Borel

**Affiliations:** ^1^Department of Biomedical Sciences, Quillen College of Medicine, East Tennessee State UniversityJohnson City, TN, USA; ^2^Department of Pathobiology, Institute of Veterinary Pathology, University of ZurichZurich, Switzerland

**Keywords:** *Chlamydia pecorum*, chlamydial persistence, chlamydial stress response, stressed chlamydiae, porcine epidemic diarrhea virus

## Abstract

Chlamydiae may exist at the site of infection in an alternative replicative form, called the aberrant body (AB). ABs are produced during a viable but non-infectious developmental state termed “persistence” or “chlamydial stress.” As persistent/stressed chlamydiae: (i) may contribute to chronic inflammation observed in diseases like trachoma; and (ii) are more resistant to current anti-chlamydial drugs of choice, it is critical to better understand this developmental stage. We previously demonstrated that porcine epidemic diarrhea virus (PEDV) co-infection induced *Chlamydia pecorum* persistence/stress in culture. One critical characteristic of persistence/stress is that the chlamydiae remain viable and can reenter the normal developmental cycle when the stressor is removed. Thus, we hypothesized that PEDV-induced persistence would be reversible if viral replication was inhibited. Therefore, we performed time course experiments in which Vero cells were *C. pecorum*/PEDV infected in the presence of cycloheximide (CHX), which inhibits viral but not chlamydial protein synthesis. CHX-exposure inhibited PEDV replication, but did not inhibit induction of *C. pecorum* persistence at 24 h post-PEDV infection, as indicated by AB formation and reduced production of infectious EBs. Interestingly, production of infectious EBs resumed when CHX-exposed, co-infected cells were incubated 48–72 h post-PEDV co-infection. These data demonstrate that PEDV co-infection-induced chlamydial persistence/stress is reversible and suggest that this induction (i) does not require viral replication in host cells; and (ii) does not require *de novo* host or viral protein synthesis. These data also suggest that viral binding and/or entry may be required for this effect. Because the PEDV host cell receptor (CD13 or aminopeptidase N) stimulates cellular signaling pathways in the absence of PEDV infection, we suspect that PEDV co-infection might alter CD13 function and induce the chlamydiae to enter the persistent state.

## Introduction

The *Chlamydiaceae* are Gram-negative, obligate intracellular bacteria that cause a large spectrum of diseases in both humans and agriculturally important animals. For example, *Chlamydia suis*, *C. abortus*, *C. pecorum* and *C. psittaci* cause syndromes in swine ranging from conjunctivitis to abortion (Pospischil et al., 2010). Asymptomatic chlamydial infections are also common in pigs and can render them more susceptible to other infections (reviewed in Schautteet and Vanrompay, 2011). Related chlamydial species, such as *C. trachomatis*, also cause medically important conditions, like trachoma, in humans. Though chlamydial infections can cause acute symptoms, they are most associated with chronic inflammation and scarring, which can result in significant host tissue damage (Schachter, 1999). However, to play a causative role in chronic diseases, chlamydiae would need to persist within infected cells/tissues for extended periods of time. How the organisms maintain long-term host infection is a central question in chamydial biology.

Chlamydiae are characterized by a complex developmental cycle, in which they alternate between a metabolically less-active, infectious form (the elementary body or EB), and a more metabolically active, replicative form (the reticulate body or RB). Upon host cell infection, the EB converts into an RB, which grow and divide within a cytoplasmic, membrane-bound inclusion. After several rounds of division, RBs then convert back into infectious EBs, which are released from the host cell (reviewed in Wyrick, 2000). The third developmental stage, variously termed persistence or the chlamydial stress response, is defined as a viable but non-cultivable state. Persistent/stressed RBs are enlarged, irregularly shaped and non-dividing; these altered developmental forms are called aberrant bodies (ABs). A variety of stressors induce chlamydial persistence/stress: these include IFN-γ exposure; glucose, iron, and amino acid depravation; penicillin G exposure; and heat shock (reviewed in Hogan et al., 2004; Wyrick, 2010; Schoborg, 2011). Interestingly, chlamydiae can remain in the persistent/stressed state in culture for up to 9 months (Galasso and Manire, 1961). Once the stressor is removed, persistent/stressed chlamydiae can reenter normal development and produce infectious EBs, which suggests that persistent/stressed chlamydiae may serve as a long-term reservoir for pro-inflammatory chlamydial antigens and/or infectious organisms (reviewed in Hogan et al., 2004; Wyrick, 2010). Although this hypothesis has not yet been directly tested, there is significant evidence that the persistent/stressed state can occur during *in vivo* infection. For example, ABs have been observed in tissues isolated from *C. suis*-infected swine (Pospischil et al., 2009), *C. muridarum*-infected mice (Rank et al., 2011; Phillips-Campbell et al., 2012) and *C. trachomatis*-infected humans (Nanagara et al., 1995).

Mixed infections are prevalent in both humans and other animals and may alter pathogenesis of one, or more, of the agents involved (recently reviewed in Debiaggi et al., 2012; Stelekati and Wherry, 2012; Alizon et al., 2013). Unfortunately, typical experimental systems exploring the interaction between a single pathogen and cell type do not accurately reflect host-multiple pathogen interplay observed *in vivo*. Therefore, it seems worthwhile to test interactions between multiple pathogenic microorganisms in simplified cell culture systems. In one such system, chlamydiae within Herpes Simplex Virus (HSV) super-infected genital epithelial cells entered the persistent/stressed state (Deka et al., 2006, 2007) via a mechanism distinct from previously characterized models of chlamydial persistence (Vanover et al., 2008). More recent data indicate that HSV glycoprotein D/host nectin-1 interaction restricts *C. trachomatis* development (Vanover et al., 2010) by an as yet incompletely characterized mechanism involving increased host cellular oxidative stress (Prusty et al., 2012). Both HSV (Deka et al., 2006, 2007; Vanover et al., 2010) and Human Herpes Virus 6 (Prusty et al., 2012) induce persistence by mechanisms that are independent of productive virus infection, but require host cell attachment and/or uptake of the virus by the host cell. As we are interested in chlamydial and viral swine pathogens, our group established a culture model of porcine epidemic diarrhea virus (PEDV)/*C. pecorum* co-infection (Stuedli et al., 2005). Both *C. pecorum* and PEDV (a coronavirus) cause economically-important gastrointestinal infections in swine (Pensaert and de Bouck, 1978; Pospischil et al., 2010). PEDV super-infection of *C. pecorum*-infected Vero cells: (i) induced AB formation; and (ii) reduced chlamydial infectivity, both of which are consistent with induction of the persistence/stress response (Borel et al., 2010). Notably, herpesviruses (which are double-stranded DNA viruses) and coronaviruses (which are single-stranded RNA viruses) use different attachment/entry mechanisms, replicate in different cellular compartments, replicate their genomes via different mechanisms, and infect different host cell types. Thus, it seems unlikely that HSV and PEDV induce chlamydial persistence/stress by the same mechanism. As a first step in dissecting the mechanism by which PEDV super-infection alters chlamydial development, we tested the hypothesis that viral replication is required for the PEDV-mediated *C. pecorum* persistence/stress response.

## Materials and methods

### Host cells, chlamydiae, and viruses

Vero 76 cells (African green monkey kidney cells, CRL 1587, American Type Culture Collection) were propagated in growth medium: Minimal Essential Medium (MEM) with Earle's salts, 25 mM HEPES, without L-glutamine (GIBCO, Invitrogen, Carlsbad, CA) but with 10% fetal calf serum (FCS) (BioConcept, Allschwil, Switzerland), 4 mM GlutaMAX-I (200 mM, GIBCO) and 0.2 mg/ml gentamycin (50 mg/ml, GIBCO). For infection experiments, Vero cells were seeded on round glass coverslips (13 mm diameter, Thermo Fisher Scientific, Cambridge, UK) at 2 × 10^5^/well in growth medium without gentamycin. *Chlamydia pecorum* 1710S (an intestinal swine isolate kindly provided by J. Storz, Baton Rouge, Louisiana, LA, USA) was used in this study. Stocks of *C. pecorum* were propagated in HEp-2 cell monolayers, purified and stored at −80°C in sucrose-phosphate-glutamate (SPG) medium as described (Borel et al., 2010). An MOI of 1 of *C. pecorum* was used for all mono-infection and mixed-infection experiments. Both *C. pecorum* and *C. abortus* development is altered by PEDV co-infection (Stuedli et al., 2005; Borel et al., 2010), but *C. pecorum* was chosen for this study because it is more sensitive to PEDV co-infection than is *C. abortus* (Borel et al., 2010). Ca-PEDV strain CV777 (kindly provided by M. Ackermann, Institute of Virology, University of Zurich) was propagated as previously described (Hofmann and Wyler, 1988), but without antibiotics for culturing the cells and for viral stock preparation. The virus stock (1 × 10^5.5^ TCID50/ml) was used undiluted for mixed-infections.

### Mixed-infection protocol

**Figure 1:**
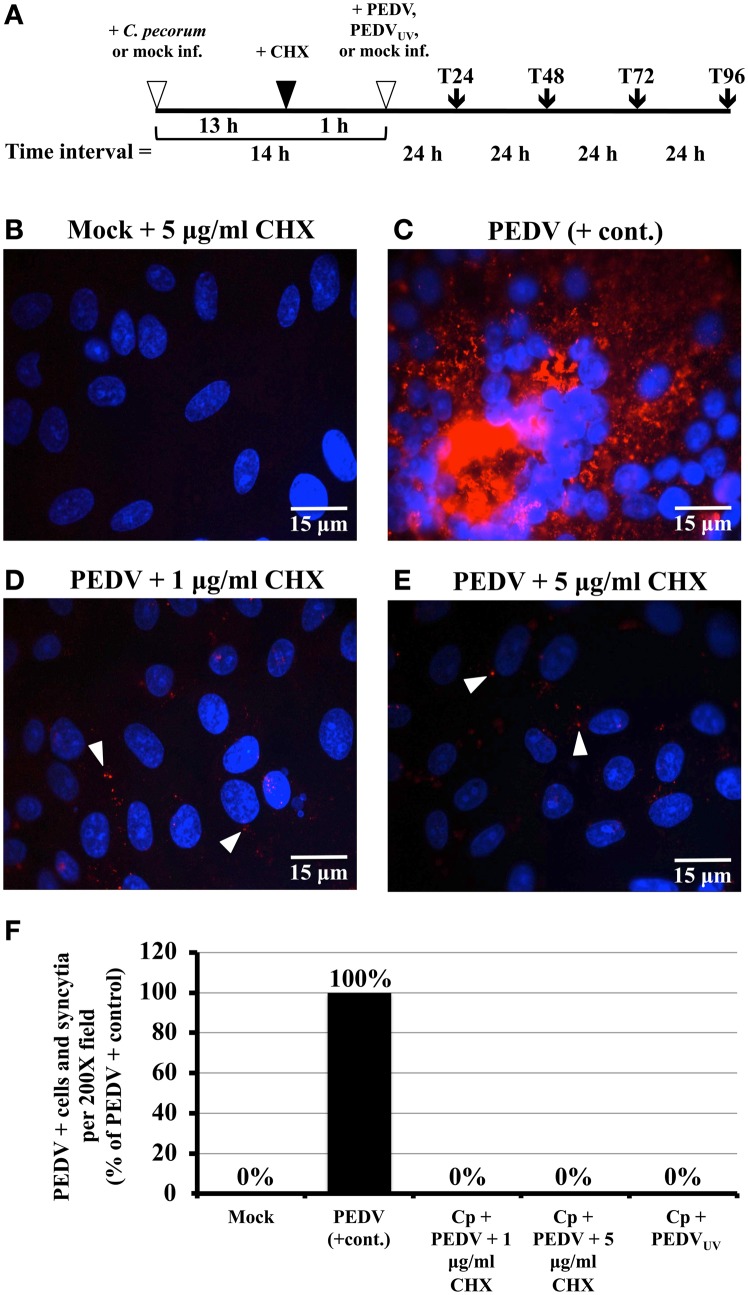
**Exposure to cycloheximide (CHX) and UV inactivation inhibits PEDV replication in Vero cells. (A)** Simplified diagram of the co-infection procedure. At the start of each experiment, replicate Vero cell cultures on coverslips were either mock- or *C. pecorum*-infected. Fourteen hours later, they were mock-, PEDV- or PEDV_UV_-infected, as appropriate. In some experiments, cells were CHX-exposed from 1 h prior to viral infection to the end of the experiment. Regardless, all cells were refed with fresh growth medium (+ or − CHX as appropriate) after PEDV infection (T0). Samples were harvested for various analyses at 24 (T24), 48 (T48), 72 (T72) or 96 (T96) hpvi. **(B–E)** Vero cells were pre-exposed to either 1 μg/ml CHX or 5 μg/ml CHX, as indicated. One hour later, cultures were either PEDV-infected (PEDV) or mock-infected (Mock). Cultures were incubated for 48 hpvi, fixed, and immunolabeled with anti-PEDV M (red) and DAPI (blue). Representative fields at 1000× magnification are shown with 15 μm scale bars. White arrows indicate anti-PEDV M punctate staining, as discussed in the text. **(F)** Vero cells were mock-infected (Mock), mono-infected with PEDV (PEDV + cont.), infected with *C. pecorum* and PEDV (Cp + PEDV), or infected with *C. pecorum* and PEDV_UV_ (Cp + PEDV_UV_) as in **(A)**. In some cases, the cells were pre-exposed to 1 μg/ml CHX or 5 μg/ml CHX, as indicated. At 24 hpvi, cells were fixed, and labeled with anti-PEDV M (red) and DAPI (blue). Twenty random 200× magnification fields were examined on each coverslip; PEDV M protein positive single cells and syncytia were counted for each field, added together, and the average total number/field calculated. The PEDV positive control was set at 100% and the average counts obtained for the other samples were used to calculate % of the positive control. The % positive control values obtained were then plotted on the Y-axis; sample identity is shown below the X-axis.

**Figure 2:**
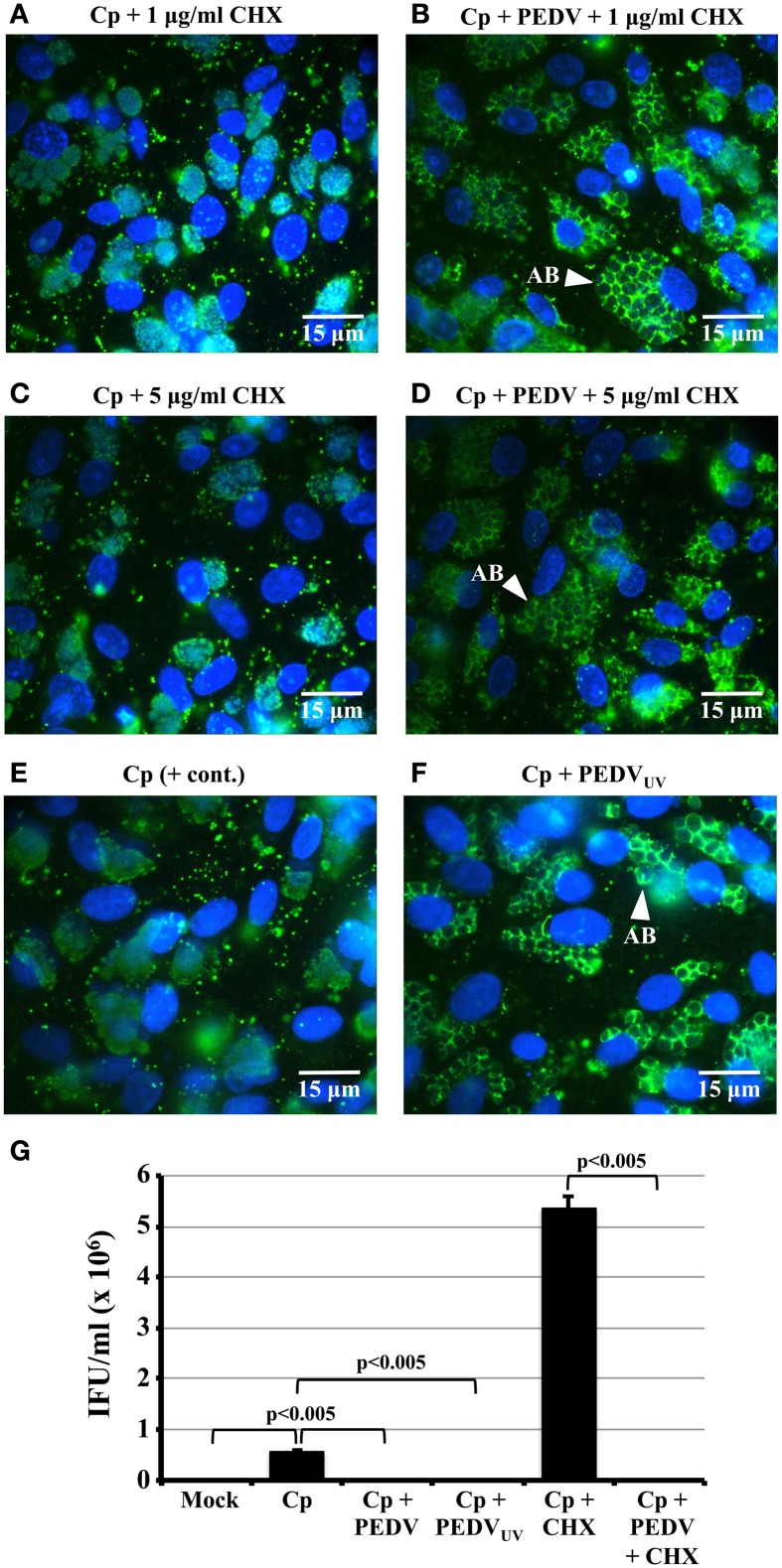
**PEDV viral replication is not required for PEDV interference with *C. pecorum* development. (A–F)** Vero cells were mock-infected (Mock), mono-infected with *C. pecorum* (Cp), infected with *C. pecorum* and PEDV (Cp + PEDV), or infected with *C. pecorum* and PEDV_UV_ (Cp + PEDV_UV_) as diagrammed in Figure 1A. In some cases, the cells were pre-exposed to either 1 or 5 μg/ml CHX to prevent PEDV replication, as indicated. At 24 hpvi, replicate coverslips were fixed, and labeled with anti-chlamydial LPS (green) and DAPI (blue) or anti-PDV M protein and DAPI. Quantification of PEDV positive cells and syncytia from this experiment is shown in Figure 1F. Representative fields at 1000× magnification are shown with 15 μm scale bars. White arrows indicate anti-LPS-staining AB. **(G)** Replicate cultures from the co-infections in **(A–F)** were subjected to sub-passage titer analysis. Some monolayers were pre-exposed to 5 μg/ml CHX (+ CHX samples) as previously described. Inclusion counts were used to calculate inclusion forming units (IFU)/mL (Y-axis). The average from three biologic replicates ± s.e.m. is shown and the data are representative of three independent experiments. Statistical comparisons are indicated by brackets and were all significant to *p* ≤ 0.005.

Mixed-infections were performed essentially as described (Borel et al., 2010). Briefly, replicate Vero cells on coverslips were divided into four groups: mock-infected, *C. pecorum*-infected, PEDV-infected, and *C. pecorum*/PEDV co-infected. For co-infections, cell monolayers were first infected with *C. pecorum* at 1MOI. After centrifugation for 1 h at 1000 × g and 25°C, the infected monolayers were subsequently incubated for 14 h at 37°C in growth medium without gentamycin. At time 0 (T0), all cell monolayers used for either mixed-infection or PEDV mono-infection were PEDV-infected (1 × 10^5.5^ TCID50), whereas for chlamydial mono-infections and mock-infections, only growth medium was applied. In some experiments, an equal volume of UV-inactivated PEDV (PEDV_UV_) was used. After viral infection, all cells were centrifuged again, after which the inoculum was removed, the cells refed with growth medium without gentamycin, and incubated for an additional 24, 48, 72, or 96 h, depending upon the experiment (Figure 1A). Replicate samples were then subjected to immunofluorescence (IF), transmission electron microscopy (TEM), or infectious titer analysis, as appropriate. In some experiments, cycloheximide (CHX), which inhibits host cellular (Obrig et al., 1971), coronaviral (van den Worm et al., 2011) but not chlamydial (Ripa and Mårdh, 1977) protein synthesis, was added to the culture medium 1 h before PEDV infection. In these experiments, all cultures were incubated in growth medium plus either 1 or 5 ug/ml CHX from the addition time until the end of the experiment. Since both 1 and 5 μg/ml CHX inhibit PEDV replication (Figure 1) but not *C. pecorum* development (Figure 2), 5 μg/ml CHX was used in time course/recovery experiments to suppress PEDV replication.

### UV inactivation of PEDV

Five hundred microliter aliquots of PEDV stock were UV-inactivated using a UV 500 crosslinker (Amersham Biosciences, Little Chalfont, UK), as described (Deka et al., 2007), using a total UV dose of 4 J/cm^2^. Similar UV doses have been used to inactivate other coronaviruses (Darnell et al., 2004). UV-inactivated PEDV stocks were unable to induce PEDV M protein positive staining syncytium formation when inoculated into Vero cell monolayers, even after a 48 h incubation period (data not shown). In contrast, control cultures infected with replication competent PEDV showed PEDV positively staining single cells and syncytia in this same time period. These data indicate that the PEDV stocks were successfully UV-inactivated.

### Immunofluorescence assays (IFA)

Infected monolayers on coverslips were methanol fixed and IFA stained immediately post-fixation as described (Borel et al., 2010). PEDV-infected single cells (and syncytia) were detected using a mouse monoclonal antibody against the viral 27 kD integral membrane M protein (mcAb 204, kindly provided by M. Ackermann, Institute of Virology, University of Zurich), diluted 1:4 in PBS plus 1% BSA, and a 1:500 diluted Alexa Fluor 594-conjugated goat anti-mouse secondary antibody (Molecular Probes, Eugene, USA). Chlamydial inclusions were labeled with a *Chlamydiaceae* family-specific mouse monoclonal antibody directed against lipopolysaccharide (LPS, Clone ACI-P, Progen, Heidelberg, Germany), and a 1:500 diluted Alexa Fluor 488-conjugated secondary goat anti-mouse antibody (Molecular Probes). Host and chlamydial DNA were labeled using 1 μg/ml 4',6-Diamidin-2'-phenylindoldihydrochlorid (DAPI, Molecular Probes). As both primary antibodies were of mouse origin, PEDV and chlamydia-specific labeling were performed on separate, duplicate coverslips. Coverslips were mounted inverted on glass slides using Immumount (Shandon, Pittsburgh, USA). Samples were examined under oil immersion at 1000× magnification using a Leica DMLB fluorescence microscope (Leica Microsystems, Wetzlar, Germany). Fluorescence photomicrographs were captured with the BonTec measuring and archiving software (BonTec, Bonn, Germany).

### Transmission electron microscopy (TEM)

Coverslips were fixed in 2.5% glutaraldehyde (Electron Microscopy Sciences, Ft. Washington, USA) for 1 h, and processed for embedding in epoxy resin (Borel et al., 2010). Ultrathin sections (80 nm) were mounted on gold grids (Merck Eurolab AG, Dietlikon, Switzerland), contrasted with uranyl acetate dihydrate (Fluka), and lead citrate (lead nitrate and tri-natrium dihydrate; Merck Eurolab AG). Fixed and counterstained gold thin sections were examined at 7000× magnification with a Tecnai 10 (FEI) transmission electron microscope at 60–80 kV in the Quillen College of Medicine TEM Core Facility.

### Chlamydial titration by subpassage

Depending upon the experiment, monolayers were scraped into 1 ml of cold growth medium at 24, 48, or 72 h post viral infection (hpvi). Infected host cell lysates were harvested and sub-passaged on fresh Vero cell monolayers in triplicate as described (Borel et al., 2010). Fixation and staining with DAPI and anti-chlamydial LPS was performed as described above. The number of inclusions in 20 random microscopic fields per sample was determined using a Leica fluorescence microscope at 200× magnification. Triplicate coverslips were counted and the counts averaged for each coverslip. The number of inclusion-forming units (IFU) in the undiluted inoculum was then calculated and expressed as IFU per ml inoculum as described (Deka et al., 2006).

### Statistical analyses

With the exception of the TEM experiments, all experiments were repeated three times independently. TEM experiments were performed twice. Statistical analyses for chlamydial titrations were performed using Microsoft Excel. The IFU/ml value for each biologic replicate is the mean of three determinations (3 replicate titer coverslips). All plotted IFU/ml values are averages of three biologic replicates ± standard error of the mean (SEM). These were compared using a 2-sample *t*-test for independent samples and *p* values of ≤0.05 were considered significant.

## Results

### PEDV replication in vero cells is inhibited by UV-inactivation and CHX exposure

Although there is a newly developed system for PEDV RNA recombination and gene replacement (Li et al., 2013a), there is currently no available system for complementing/propagating PEDV replication-deficient mutants. As a result, we could not use such mutants to address our hypothesis. Therefore, we used UV inactivation and host cell CHX pre-treatment to inhibit PEDV replication in co-infected cells. A similar approach was used to determine that HSV replication was required for chlamydial persistence/ stress induction (Deka et al., 2007). CTX was chosen because it inhibits host mammalian cell (Obrig et al., 1971) and coronaviral (van den Worm et al., 2011) protein synthesis, but not that of chlamydiae (Ripa and Mårdh, 1977). UV light has been used to inactivate other coronaviruses (Darnell et al., 2004) and is widely used to inactivate virions without altering their ability to bind and enter host cells. To test the efficacy of these inactivation methods for PEDV, Vero monolayers were pre-exposed for 1 h to growth medium plus: (i) 1 μg/ml CHX; or (ii) 5 μg/ml CHX. CHX was also added to the PEDV inoculum and to the culture medium after infection. Cultures were incubated 48 h post-infection (hpi) and then fixed, IFA stained to detect PEDV antigens and examined microscopically for PEDV-positive cells and syncytia (Figures 1B–E). During viral replication, PEDV antigen-positive cells and syncytia with up to 50–100 nuclei are observed (Hofmann and Wyler, 1988; Borel et al., 2010) and, because coronaviruses replicate in the host cell cytoplasm, anti-M protein staining is primarily cytoplasmic (Figure 1C; Borel et al., 2010). Thus, these characteristics can be used to determine whether PEDV productive replication has occurred during an experiment. As expected, positively staining single infected cells and syncytia were readily detectable in PEDV-infected cultures (Figure 1C). Addition of either 1 or 5 μg/ml CHX prior to PEDV infection eliminated PEDV antigen-positive single cells and syncytium formation (Figures 1D,E). Likewise, Vero monolayers infected with UV-inactivated PEDV (PEDV_UV_) contained neither M protein cytoplasmically-positive cells nor syncytia (data not shown). These data indicate that host cell pre-exposure to CHX and UV-inactivation both inhibit PEDV replication. Though strong cytoplasmic anti-M protein staining is not observed in PEDV_UV_-infected or CHX-pre-exposed cultures, small dots of anti-M protein immunostaining are observed in these cultures (Figures 1D,E, white arrows). These anti-M foci are not observed in uninfected cultures (Figure 1B) and are unlikely to be background staining. As the M protein is an abundant structural component of coronavirus particles, these foci are most likely immunolabeled PEDV virions that have bound to and/or entered into host cells. These data suggest that CHX and UV inactivation inhibit PEDV replication but not viral attachment/entry.

### PEDV replication is not required to alter *C. pecorum* development

To determine whether PEDV replication is required to alter *C. pecorum* development, Vero monolayers were infected first with *C. pecorum* and later with PEDV, or mock-infected, as described above. In some replicates (Figures 2A–D), cells were pre-exposed to 1 or 5 μg/ml CHX before viral infection. In others, cells were co-infected with PEDV_UV_ in the absence of CHX (Figure 2F). As expected, neither mock- nor PEDV singly-infected cells stained with anti-LPS (Figures S1A,D). IFA staining with anti-LPS revealed normal inclusions in *C. pecorum*-infected control cells (Figure 2E, Figures S1B,C). In contrast, inclusions within *C. pecorum*/PEDV co-infected cells contained anti-LPS tagged, greatly enlarged AB (Figures S1E,F, white arrows), as previously reported (Borel et al., 2010). Exposure to either 1 μg/ml (Figure 2A) or 5 μg/ml CHX (Figure 2C) had no effect on inclusion size, morphology or anti-LPS staining intensity compared to a *C. pecorum*-infected control in the absence of CHX (Figure 2E). Notably, AB were readily apparent in *C. pecorum*/PEDV co-infected cultures in the presence of CHX (Figures 2B,D, white arrows). Co-infection with PEDV_UV_ similarly induced AB formation in the absence of CHX (Figure 2F, white arrow). Infectious titer analysis on replicate cultures indicated that co-infection with PEDV, PEDV_UV_ or PEDV in the presence of CHX significantly decreased infectious titer compared to either the *C. pecorum* alone or *C. pecorum* + CHX controls, as appropriate (Figure 2G). Finally, anti-PEDV IFA of replicate coverslips indicated that the UV inactivation and CHX-exposure completely eliminated PEDV (+) single cells and syncytia (Figure 1F), as previously observed (Figures 1D,E). These data indicate that PEDV replication is not required for co-infection induced persistence/stress induction and suggest that PEDV binding/entry may be sufficient to induce this effect.

### The PEDV-induced *C. pecorum* developmental cycle alteration is reversible

**Figure 3:**
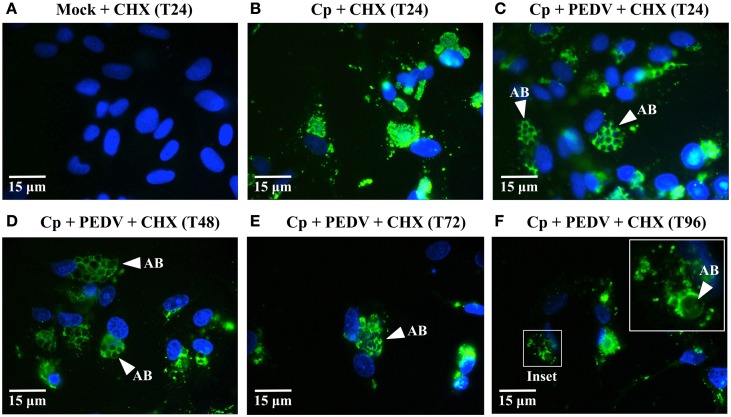
**ABs are present in co-infected cells for up to 96 hpvi when PEDV replication is blocked. (A–F)** Vero cells were mock-infected (Mock), mono-infected with *C. pecorum* (Cp), or co-infected with *C. pecorum* and PEDV (Cp + PEDV) as diagrammed in Figure 1A. All cultures were pre-exposed to 5 μg/ml CHX starting at 1 h before viral infection to prevent PEDV replication, as indicated. At 24 hpvi (T24), 48 hpvi (T48), 72 hpvi (T72), and 96 hpvi (T96), replicate coverslips were fixed and labeled with anti-chlamydial LPS (green) and DAPI (blue). Replicate coverslips were also stained for PEDV M protein to confirm suppression of viral replication; results similar to those in Figure 1F were obtained (data not shown). Representative fields from anti-LPS/DAPI stained coverslips are shown at 1000× magnification with 15 μm scale bars. White arrows indicate anti-LPS-stained AB. In **(F)**, the inset shows a higher magnification view of an inclusion at 96 hpvi (white box).

**Figure 4:**
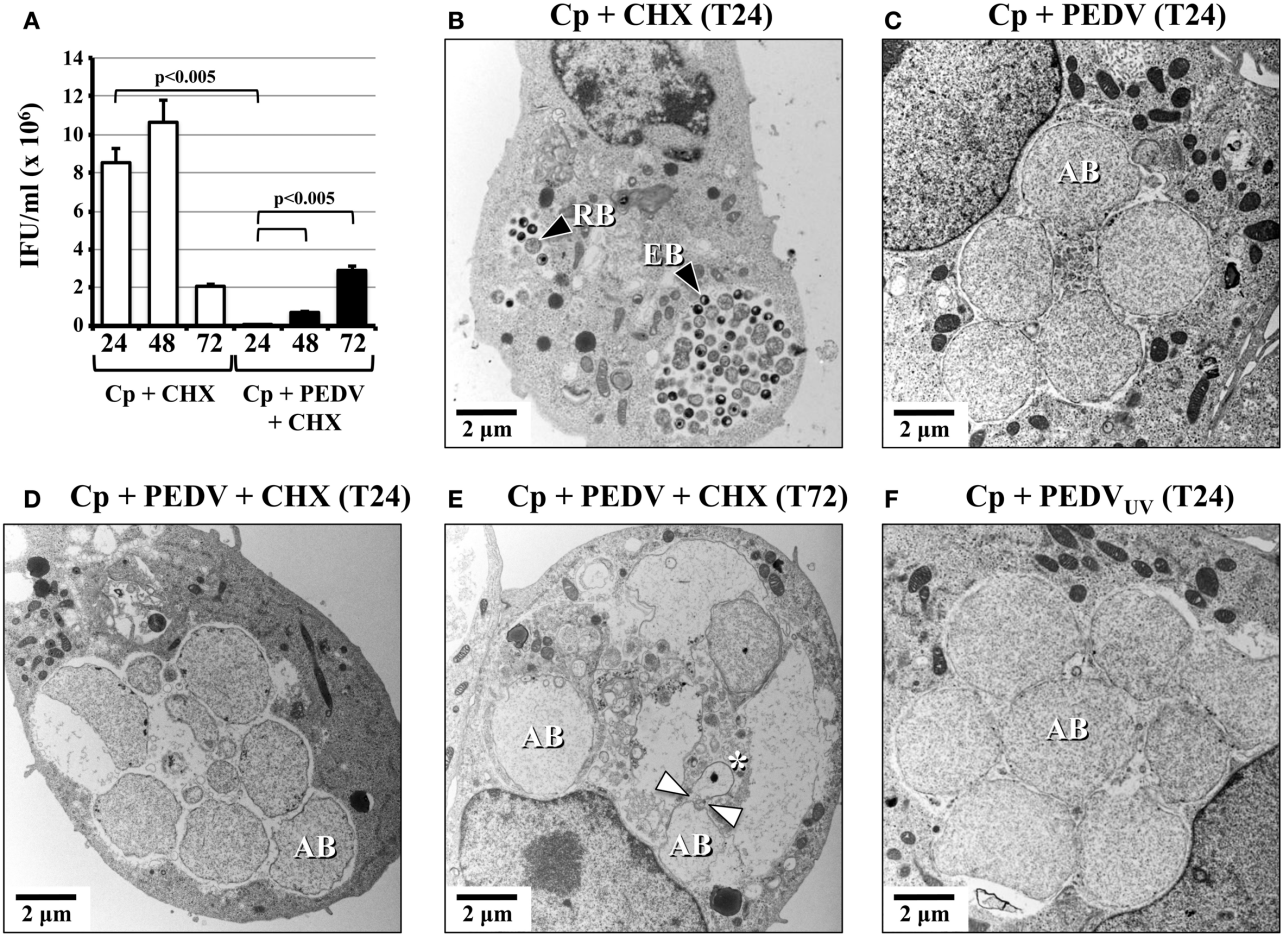
**The PEDV-induced developmental cycle disruption is reversible. (A)** Vero cells were mock-infected (Mock; data not shown), mono-infected with *C. pecorum* (Cp), or co-infected with *C. pecorum* and PEDV (Cp + PEDV) as diagrammed in Figure 1A. Cultures (+CHX) were pre-exposed to 5 μg/ml CHX starting at 1 h before viral infection to prevent PEDV replication, as described in Figure 1A. At 24 hpvi (T24), 48 hpvi (T48), and 72 hpvi (T72), replicate coverslips were used for sub-passage titer analysis. Inclusion counts were used to calculate inclusion forming units (IFU)/mL (Y-axis). The time hpvi and sample are shown below the X-axis. The average from three biologic replicates ± s.e.m. is shown; these data are representative of three independent experiments. Statistical comparisons are indicated by brackets and all were significant to *p* <0.005. **(B–F)** Vero cells were mock-infected (Mock; not shown), infected with *C. pecorum* (Cp), infected with *C. pecorum* and PEDV (Cp + PEDV), or infected with *C. pecorum* and PEDV_UV_ (Cp + PEDV_UV_), as shown in Figure 1A. Some cultures (+CHX) were pre-exposed to 5 μg/ml CHX starting at 1 h before viral infection. At 24 hpvi (T24) and 72 hpvi (T72), replicate coverslips were fixed and processed for transmission electron microscopy. Representative photomicrographs at 7000× magnification are shown, scale bars are 2 μm. RB (RB-black arrow), EB (EB-black arrow), and AB are indicated. A white asterisk and double white arrows indicate a small AB with a condensed nucleoid that may be “budding” from an adjacent AB **(E)**.

One hallmark of the non-infectious but viable state is that it is reversible—if the stressor is removed, the chlamydiae re-enter normal development and infectious progeny are produced (reviewed in Hogan et al., 2004; Wyrick, 2010; Schoborg, 2011). Since CHX prevented PEDV replication in host cells but did not interfere with persistence/stress induction, we reasoned that any PEDV particle components responsible for this effect might eventually be degraded (and persistence/stress subsequently “reversed”) if co-infected cells were kept under continuous CHX exposure to prevent viral replication. A similar approach demonstrated that HSV-induced persistence was also reversible (Vanover et al., 2010). Therefore, we co-infected and CHX-exposed replicate Vero cultures as previously described, except that coverslips were collected at 24, 48, 72, and 96 hpvi (Figures 3A–F). IFA revealed anti-LPS staining ABs in CHX-exposed, PEDV co-infected cultures out to 96 hpvi (Figures 3C–F). In contrast, inclusions in *C. pecorum* + CHX cultures did not contain visible ABs (Figure 3B). Notably, at 72 and 96 hpvi (Figures 3E,F), co-infected cultures contained fewer host cell nuclei (and inclusions) than did cultures harvested at earlier times (Figures 3A–D). Because reentry into normal development and production of infectious EBs is one possible explanation for the observed host cell and inclusion loss, we performed infectious titer assays on replicate samples (Figure 4A). As previously observed, PEDV co-infection significantly reduces infectivity at 24 hpvi, compared to that in *C. pecorum* control cultures at the same time. Importantly, production of infectious EBs from PEDV co-infected, CHX-exposed cultures is significantly increased at 48 and 72 hpvi, compared to co-infected samples collected at 24 hpvi. These data indicate that the PEDV-induced loss of chlamydial infectivity is reversible within 48 hpvi if continued viral replication is inhibited.

### PEDV co-infection induces *C. pecorum* AB morphologically indistinguishable from persistent/stressed organisms

Persistent/stressed chlamydiae have a striking ultrastructural appearance (reviewed in Hogan et al., 2004; Wyrick, 2010; Schoborg, 2011). The enlarged organisms observed by IFA in co-infected cells resemble ABs, but the AB morphology is best observed by TEM. Therefore, we performed electron microscopy on replicate samples from the time course infection/CHX-exposure experiment described above. Unsurprisingly, inclusions in *C. pecorum*-infected cells exposed to CHX contained normal RBs and EBs (Figure 4B, black arrows). Greatly enlarged, misshapen RBs (i.e., ABs similar to those observed in other persistence/stress tissue culture models) were present in co-infected cells (Figure 4C) at 24 hpvi as previously observed (Borel et al., 2010). Co-infected, CHX-exposed samples at both 24 and 72 hpvi (Figures 4D,E) and in cells co-infected with PEDV_UV_ at 24 hpvi (Figure 4F) also contain primarily ABs. Interestingly, smaller ABs with condensed, darkly staining nucleoids, were observed at later times post-PEDV infection (Figure 4E, white asterisk), some of which appeared to be in the process of “budding” from larger ABs (Figure 4E, white arrows). Thus, chlamydiae within PEDV co-infected cells have the typical persistent/stressed AB ultrastructure regardless of whether or not viral replication is prevented by CHX-exposure.

## Discussion

Taken together, these and previously published (Borel et al., 2010) data definitively demonstrate that PEDV co-infection induces the *C. pecorum* persistence/stress response. Both anti-LPS IFA and electron microscopic examination indicates the presence of grossly enlarged, electronlucent ABs (Figure S1, Figure 4 and Borel et al., 2010), which is consistent with the interrupted RB cytokinesis observed during persistence/stress (Matsumoto and Manire, 1970). Chlamydiae within co-infected cells are viable (as shown by the ability to recover infectivity by 48 hpvi if viral replication is inhibited by CHX-exposure; Figure 4A), but non-infectious (as shown by reduced chlamydial titer immediately post-co-infection; Figure 2G and Borel et al., 2010). Published data indicate that: (i) recovery from penicillin-exposure takes 10–20 h after drug removal; and (ii) replicative RBs may “bud” from ABs to reenter the productive developmental cycle (Skilton et al., 2009). We observe similar recovery timing, in that infectious EBs are not observed until 48 h after PEDV infection and CHX addition (Figure 4A). Interestingly, at late times post-PEDV/CHX addition, we detect smaller ABs with condensed nucleoids, some of which appear to be budding from larger ABs (Figure 4E). However, the presence (or absence) of replicative RBs “budding” from ABs in co-infected cells can only be confirmed by time lapse photography, similar to that published by Skilton et al. Though we do not currently have access to the necessary equipment, it might prove interesting to perform such analyses in the future to determine whether AB to RB “budding” is a general characteristic of the transition from persistent/stress to normal development regardless of the stressor used.

Chlamydiae in co-infected cells enter the persistent/stressed state regardless of whether PEDV replication is inhibited by CHX-exposure or UV-inactivated virions are used for co-infection (Figures 2, 4D–F). Control experiments show that PEDV replication ceases under these conditions (Figures 1B–F), demonstrating that PEDV replication is not required to induce the persistence/stress response. These data have several important implications. First, PEDV-induced *C. pecorum* persistence is unlikely to be a byproduct of host resource consumption by the replicating virus. This is an important issue because host cellular nutrient deprivation can cause developing chlamydiae to enter the persistent/stressed state (reviewed in Hogan et al., 2004; Wyrick, 2010; Schoborg, 2011). Second, UV light inactivates RNA viruses by damaging the genome, which prevents genomic replication and subsequent events (like viral gene expression and assembly). As a result, it seems more likely that an early event in the PEDV replication cycle, such as host cell attachment or entry, triggers this response. If so, the initiating molecule is most likely to be a physical component of the PEDV particle. Third, these data also suggest that host proteins synthesized in response to PEDV co-infection, like cytokines or type 1 interferons, are also unlikely to be involved. Notably, Luminex bead-based ELISA experiments indicate that neither TNF-α, nor IFN-γ are detected in co-infected culture supernatants, though IL-6 is observed (data not shown). Thus, PEDV-induced *C. pecorum* persistence/stress is unlikely to be mediated by the cytokines currently known to induce this response (reviewed in Hogan et al., 2004; Wyrick, 2010; Schoborg, 2011).

If a PEDV virion component does, indeed, influence the *C. pecorum* developmental cycle, which is the most likely component involved? The coronavirus literature suggests several likely candidates, one of which is the viral envelope S glycoprotein. PEDV attachment and entry are initiated when the S protein binds to aminopeptidase N (APN or CD13) on the host cell surface (Li et al., 2007; Nam and Lee, 2010). CD13 is a 150- to 160-kDa type II glycoprotein that has peptidase activity and is expressed by epithelial cells in the kidney, intestine and respiratory tract (Wentworth and Holmes, 2001). CD13 is a known modulator of signal transduction and cell motility (Petrovic et al., 2007), and regulates TNF-α-induced apoptosis in neutrophils by inhibiting TNFRI shedding (Cowburn et al., 2006). CD13 also co-localizes with FcγRI (a receptor for immunoglobulin Fc) on the monocytic cell membrane, suggesting it may act as a regulator of FcγRI signaling (Mina-Osorio and Ortega, 2005). The SARS coronavirus (SARS CoV) S protein is also a pathogen associated molecular pattern (PAMP) that signals through the host Toll-like receptor 2 (TLR-2) to stimulate IL-8 production from human macrophages (Dosch et al., 2009). Finally, mouse hepatitis virus (MHV) and SARS-CoV S proteins increase endoplasmic reticulum (ER) stress in murine L fibroblasts (Versteeg et al., 2007).

Another known “bio-active” coronavirus virion component is the single-stranded RNA (ssRNA) genome, which like S protein, would be present in cells that are either infected with PEDV_UV_ or infected with replication-competent PEDV but pre-exposed to CHX. Single-stranded viral genomic RNAs (ssRNAs) are strong activators of TLR7 and TLR8, which subsequently activate diverse cellular processes, including pro-inflammatory and regulatory cytokine production (reviewed in Cervantes et al., 2012). Recently, GU-rich RNA fragments derived from the SARS-CoV ssRNA genome were shown to activate TNF-α, IL-6, and IL-12 release from murine RAW264.7 cells in culture via TLR7 and TLR8 activation. These RNAs can also cause fatal acute lung injury in mice in the absence of infectious virions (Li et al., 2013b). Thus, contact with PEDV S protein and/or genomic RNA from incoming virions can profoundly alter host cell physiologic processes. Whether or not such perturbations subsequently influence chlamydial development is currently unknown, but is a question we are very interested in answering.

As mentioned above, CHX-exposure and PEDV_UV_ infection experiments both suggest that neither *de novo* synthesized host proteins nor *de novo* produced viral components are required for PEDV-induced *C. pecorum* persistence/stress induction. However, a recent study suggests that low amounts of SARS-CoV and murine hepatitis virus (MHV) RNA synthesis can occur even when viral protein synthesis is inhibited with CHX (van den Worm et al., 2011). It is also possible that low level viral RNA expression and/or synthesis of PEDV proteins occurs in our system even when replication is inhibited by CHX or by virion UV inactivation. Though unlikely, we also have to consider the possibility that a PEDV product produced during replication (rather than a viral particle component) might initiate the observed effects on chlamydial development. The PEDV ORF3 protein is one candidate with the potential to profoundly influence the host cellular internal environment. PEDV ORF3 is a member of an increasingly large group of viral proteins called “viroporins” and has potassium channel activity when etopically expressed in either *Xenopus* oocytes or *Sacchromyces cerevisiae* (Wang et al., 2012). Another coronaviral replication product with significant host cell effects is double-stranded RNA (dsRNA), which is produced during viral genome replication (reviewed in Hagemeijer et al., 2012). Double-stranded RNA is a potent activator of both TLR3 and cytoplasmic Rig-like receptors (RLRs), which can activate IFN-β production and anti-viral host cellular responses (reviewed in Kawai and Akira, 2008). It is, therefore, possible that low-levels of dsRNA could alter chlamydial development by activating other host anti-pathogen responses. Alternatively, toxic effects of a PEDV protein, like ORF3, on the host cell could produce a similar result. Because our data indicate that PEDV replication proteins/RNA are less likely candidates, we will first examine the possible contribution of PEDV virion components, like S protein and genomic ssRNA.

While the CHX-exposure data suggest that *de novo* synthesis of host proteins in response to PEDV infection is not required to induce *C. pecorum* persistence/stress, host cells can also release preformed mediators in response to damage or infection. These molecules are called DAMPs (damage or danger associated molecular patterns) and include host proteins (like heat shock protein 60) and non-proteins [like uric acid and extracellular ATP (ATPe)] (reviewed in Piccinini and Midwood, 2010; Miyake and Yamasaki, 2012). Exposure of chlamydia-infected host cells to ATPe or adenosine (Ado) arrests the developmental cycle and reduces *C. trachomatis* infectivity, as observed during persistence/stress. However, AB formation is not observed in response to Ado (Pettengill et al., 2009). Since PEDV co-infection is a strong inducer of AB formation (Figures 2, 4, Figure S1), it is unlikely to be mediated by Ado release from co-infected cells. However, other DAMPs released from PEDV-infected cells could abort normal chlamydial development—a possibility that should be examined in the future.

Since the specific viral and/or host inducer(s) molecule is unknown, it is difficult to speculate on the mechanism by which PEDV co-infection induces *C. pecorum* persistence/stress. Prusty et al. suggest that HHV-6-induced host cellular oxidative stress activates *C. trachomatis* persistence in co-infected cells. However, antioxidant-exposure only partially reverses the observed effect, indicating that other mechanisms may also be involved (Prusty et al., 2012). Notably, SARS CoV infection increases transcription of host oxygen stress-related genes, suggesting that coronaviral infection may increase host cell oxidative stress (Hu et al., 2012). Thus, comparison of oxidative stress markers in *C. pecorum* mono-infected and co-infected cultures may also be warranted. Although we do not yet know the inducers involved or the molecular mechanism, our current data are essential to guide future studies. Regardless of the mediator involved (host or viral), its identification is likely to reveal interesting new facets of the host/chlamydial interaction and the means by which chlamydial entry into and exit from persistence/stress is regulated.

## Author contributions

Robert V. Schoborg and Nicole Borel designed the experiments, conducted all experiments and analyzed the data. Both authors contributed to drafting the manuscript and figures.

## Conflict of interest statement

The review editor Andreas Pospischil declares that, despite being affiliated to the same institution as the author Nicole Borel, the review process was handled objectively and no conflict of interest exists. The authors declare that the research was conducted in the absence of any commercial or financial relationships that could be construed as a potential conflict of interest.

## Supplementary material

Figure S1 **PEDV co-infection induces *C. pecorum* AB formation at 24 hpvi. (A–F)** Vero cells were mock-infected (Mock), mono-infected with PEDV (PEDV), mono-infected with *C. pecorum* (*C. pecorum*), or co-infected with *C. pecorum* and PEDV (Cp + PEDV) as diagrammed in Figure 1A. At 24 hpvi, replicate coverslips were fixed and labeled with anti-chlamydial LPS (green) and DAPI (blue). Representative fields at 1000× magnification are shown with 15 μm scale bars in **(A,B,D,E)**. **(C,F)** are higher magnification photos of the boxed areas in **(B,E)**. The scale bars in **(A,B,D,E)** are 15 μm; those in **(C)** and **(F)** are 5 μm. White arrows indicate anti-LPS-staining AB.
