# Supplementary figures and images for: Porcine epidemic diarrhea virus (PEDV) co-infection induced chlamydial persistence/stress does not require viral replication
Source: Front Cell Infect Microbiol. 2014 Mar 13;4:20. doi: 10.3389/fcimb.2014.00020 (PMC3952398; doi:10.3389/fcimb.2014.00020)

**A.****Mock**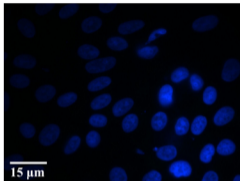**B.*****C. pecorum***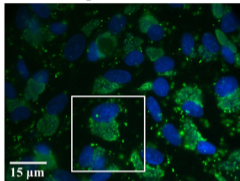**C. Enlarged from B.**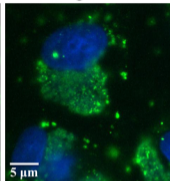**D.****PEDV**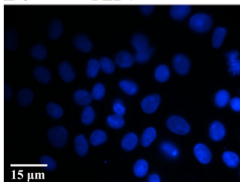**E.*****C. pecorum* + PEDV**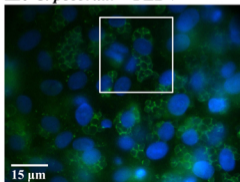**F. Enlarged from E.**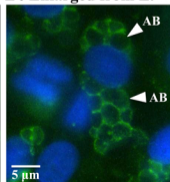

Supplement: Figure S1 — PEDV co-infection induces C. pecorum AB formation at 24 hpvi. (A–F) Vero cells were mock-infected (Mock), mono-infected with PEDV (PEDV), mono-infected with C. pecorum (C. pecorum), or co-infected with C. pecorum and PEDV (Cp + PEDV) as diagrammed in Figure 1A. At 24 hpvi, replicate coverslips were fixed and labeled with anti-chlamydial LPS (green) and DAPI (blue). Representative fields at 1000× magnification are shown with 15 μm scale bars in (A,B,D,E). (C,F) are higher magnification photos of the boxed areas in (B,E). The scale bars in (A,B,D,E) are 15 μm; those in (C) and (F) are 5 μm. White arrows indicate anti-LPS-staining AB. [file Presentation1.PDF]
